# The Bcl-2 Homology-3 Domain (BH3)-Only Proteins, Bid, DP5/Hrk, and BNip3L, Are Upregulated in Reactive Astrocytes of End-Stage Mutant SOD1 Mouse Spinal Cord

**DOI:** 10.3389/fncel.2018.00015

**Published:** 2018-01-30

**Authors:** Nathan Duval, Whitney A. Sumner, Anna G. Andrianakos, Josie J. Gray, Ron J. Bouchard, Heather M. Wilkins, Daniel A. Linseman

**Affiliations:** ^1^Biological Sciences and Knoebel Institute for Healthy Aging, University of Denver, Denver, CO, United States; ^2^Biological Sciences and Eleanor Roosevelt Institute, University of Denver, Denver, CO, United States

**Keywords:** amyotrophic lateral sclerosis (ALS), SOD1 G93A transgenic mice, Bcl-2, BH3-only proteins, glial cells, motor neurons, reactive gliosis

## Abstract

The molecular mechanisms leading to motor neuron death in amyotrophic lateral sclerosis (ALS) are unknown; however, several studies have provided evidence of a central role for intrinsic apoptosis. Bcl-2 homology-3 domain (BH3)-only proteins are pro-apoptotic members of the Bcl-2 family whose enhanced expression acts as a trigger for the intrinsic apoptotic cascade. Here, we compared the relative expression of BH3-only proteins in the spinal cord of end-stage G93A mutant SOD1 mice to age-matched wild-type (WT) mice. Large alpha motor neurons in lumbar spinal cord sections of both WT and end-stage mutant SOD1 mice stained positively for a number of BH3-only proteins; however, no discernible differences were observed in either the relative intensity of staining or number of BH3-immunoreactive motor neurons between WT and mutant SOD1 mice. On the other hand, we observed significantly enhanced staining for Bid, DP5/Hrk, and BNip3L in GFAP-positive astrocytes only in end-stage G93A mutant SOD1 spinal cord. Staining of additional end-stage G93A mutant SOD1 tissues showed specific upregulation of DP5/Hrk in lumbar spinal cord sections, but not in cerebellum or cortex. Finally, examination of protein expression using western blotting also revealed marked increases in DP5/Hrk and BNip3L in G93A mutant SOD1 lumbar spinal cord lysates compared to WT controls. The upregulation of a specific subset of BH3-only proteins, including Bid, DP5/Hrk, and BNip3L, in reactive astrocytes suggests that these proteins may execute a novel *non-apoptotic* function within astrocytes to promote ALS disease progression, thus providing a new potential target for therapeutic intervention.

## Introduction

Amyotrophic lateral sclerosis (ALS), or Lou Gehrig’s Disease, is a debilitating neuromuscular disease in which motor neuron death and retraction of motor axons from the neuromuscular junctions results in skeletal muscle weakness, atrophy, and paralysis. ALS is a rapidly progressive disorder, typically causing death within 2–5 years of onset (Pasinelli and Brown, 2006). The majority (∼90%) of ALS cases are sporadic or of unknown etiology. Mutations in Cu/Zn-superoxide dismutase (SOD1) are causative in approximately 10–20% of familial ALS cases. The G93A mutant SOD1 mouse model displays age-dependent, progressive spinal motor neuron degeneration, muscle weakness, and paralysis characteristic of ALS (Dal Canto and Gurney, 1994; Ripps et al., 1995).

Several studies have provided compelling evidence for mitochondrial oxidative stress, mitochondrial dysfunction, and intrinsic apoptosis as significant factors in the motor neuron death that underlies ALS. For instance, the G93A mutant SOD1 mouse, as well as ALS patients, show increased oxidation of mitochondrial DNA (Bogdanov et al., 2000; Warita et al., 2001). Furthermore, various antioxidants and free radical scavengers, particularly those targeted to mitochondria, attenuate motor neuron degeneration in the G93A mutant SOD1 mouse (Liu et al., 2002; Petri et al., 2006). Evidence of cytochrome c release, apoptosis inducing factor activation, reduction of X-linked inhibitor of apoptosis protein transcript levels, and caspase activation have all been demonstrated in the G93A mutant SOD1 mouse spinal cord (Li et al., 2000; Guegan et al., 2001; Inoue et al., 2003; Kirkinezos et al., 2005; Oh et al., 2006; Tokuda et al., 2007). Finally, the use of specific caspase inhibitors in this ALS model slows disease progression and delays disease onset (Li et al., 2000; Inoue et al., 2003).

Intrinsic apoptosis is regulated by the Bcl-2 family of proteins which includes both pro-survival (Bcl-2, Bcl-X_L_, Bcl-w) and pro-death proteins (multidomain; Bax and Bak and BH3-only proteins; Bim, Bad, Bik, DP5/Hrk, Puma, Noxa, Bid, and Bmf). Several studies have indicated a role for the Bcl-2 family of proteins in ALS disease pathogenesis. Bcl-2 has been shown to be downregulated in spinal cord of ALS patients and in the G93A mutant SOD1 mouse, while Bax has been shown to be upregulated (Mu et al., 1996; Ekegren et al., 1999; Vukosavic et al., 1999). Furthermore, mutant SOD1 accumulates at mitochondria where it can bind and aggregate with Bcl-2, inducing a conformational change and possibly unmasking a toxic, *pro-apoptotic* function of Bcl-2 (Pasinelli et al., 2004; Pedrini et al., 2010). Evidence for the opposing roles of Bcl-2 and Bax in ALS has been obtained through the overexpression of Bcl-2, which enhances motor neuron survival and delays onset of the disease (Kostic et al., 1997; Azzouz et al., 2000; Vukosavic et al., 2000). On the other hand, deletion of Bax by crossing Bax null mice with G93A mutant SOD1 mice produces an increase in lifespan, attenuation of reactive astrogliosis, and a delay in motor neuron degeneration (Gould et al., 2006).

BH3-only proteins act in a coordinated manner to trigger apoptosis by either inactivating Bcl-2 or inducing an active conformation of Bax (Willis and Adams, 2005; Kim et al., 2006). Several studies have suggested possible roles for BH3-only proteins in the mechanism underlying ALS disease. In ALS patient spinal cord tissues, Hrk is upregulated in comparison to age-matched control tissue (Shinoe et al., 2001). Bid has been found in its activated and truncated form, tBid, in spinal cord of G93A mutant SOD1 mice (Guegan et al., 2002). Furthermore, an increased expression of Bim has been observed in G93A mutant SOD1 mouse spinal cord and knockdown of Bim is protective against mutant SOD1 over-expression in NSC34 motor neuronal cells and in mice (Hetz et al., 2007). In a similar manner, Puma was also shown to be upregulated in spinal cord of G93A mutant SOD1 mice and subsequent knockout of Puma delayed motor neuron loss and disease onset (Kieran et al., 2007).

Here, we found an upregulation of specific BH3-only proteins within reactive astrocytes of end-stage, G93A mutant SOD1 mouse spinal cord. Based on previous studies mentioned above, BH3-only proteins appear to play a significant role in ALS pathogenesis in the context of disease induced by the mutant SOD1 gene. However, whether these proteins directly induce intrinsic apoptosis within spinal cord motor neurons is presently uncertain. Our observation that particular BH3-only proteins are upregulated specifically within reactive astrocytes of end-stage mice suggests a novel non-apoptotic role for these proteins in ALS pathogenesis.

## Materials and Methods

### G93A Mutant SOD1 Transgenic Mouse Colony

Wild type (WT) FVB females were crossed with age-matched, G93A mutant hSOD1 male animals. Pups were weaned at 21 days of age, at which time a 0.5 cm tail snip was taken from each pup. Genotyping of animals was completed by Transnetyx, Inc. (Cordova, TN, United States). G93A mutant animals have a well established disease onset of approximately 90 days, with end-stage occurring at approximately 120 days. Animals were considered end-stage and euthanized when they could no longer right themselves after being placed on their side for ten seconds. Each G93A animal was euthanized in conjunction with an age-matched WT animal. All animal procedures were performed in accordance with and under approval of the University of Denver Institutional Animal Care and Use Committee.

### Spinal Cord and Brain Dissection

Animals were euthanized by Isoflurane inhalation (Webster Veterinary) in a sealed chamber. The cortex and cerebellum were surgically dissected, and lumbar spinal cord was forced out of the spinal column using phosphate-buffered saline (PBS, pH 7.4; Gibco), a 10 ml syringe and an 18G needle (Becton Dickinson). Immediately following dissection, tissue was frozen in Optimal Cutting Temperature (OCT) compound (Tissue-Tek) in a cryo-mold over dry ice. All samples were stored at -80°C until cryo-sectioning.

### Cryo-sectioning

Frozen tissue was cut using microtome blades (Extremus, DT315X50) at -20°C. Sectioning for immunohistochemistry (IHC) was performed at 8 micron thick slices. Tissue sections cut for IHC were placed on standard superfost/plus microscope slides (Fisher Scientific, 12-550-15). All exposed tissues were covered in OCT and stored at -80°C until future use.

### Immunohistochemistry (IHC)

All antibodies and dilutions utilized for IHC are listed in **Table 1**. Primary antibodies and secondary antibodies were made up at specified dilutions in 2% BSA in PBS containing 0.2% Triton X-100. Tissue sections were fixed in 4% paraformaldehyde in PBS, followed by blocking and permeabilization in 5% BSA in PBS containing 0.2% Triton X-100. Slides were then incubated with primary antibodies overnight at 4°C in a humidified chamber. For co-staining experiments, slides were incubated with both primary antibodies simultaneously. Next, slides were washed five times in PBS at room temperature (RT; 25°C) over 30 min, and then incubated with the appropriate secondary antibody for 90 min at RT. For co-staining, slides were incubated with appropriate secondary antibodies for both primary antibodies as listed above. Subsequently, slides were washed with PBS five times over 30 min at RT, 3–5 drops of anti-quench (Sigma–Aldrich, Inc.) were added, and the slides were cover-slipped and sealed with clear nail polish. Slides were imaged with either a 40x Air or 63x Oil objective. A digital de-convolution imaging system based on a Zeiss Axioplan 2 EPI fluorescence up-right microscope platform was used. Images were captured and analyzed using Slide Book software version 4.1.0.11 and formatted in Adobe Photoshop Version 6.0. Five images were captured per slide, per condition, and per tissue, for each animal. Negative controls for non-specific (secondary-only) staining were completed by omitting primary BH3-only protein antibodies from the protocol listed above. In addition, specific blocking peptides were utilized for Bid and DP5/Hrk primary antibodies, in which the primary antibodies were pre-blocked with the blocking peptides (ProSci, Inc.) at a ratio of 1:2 (antibody:peptide) in 2% BSA with 0.2% Triton X-100/PBS and incubated on a tube rotator at 30°C and 500 rpm for one hour, after which the IHC protocol listed above was followed.

**Table 1:** Antibodies utilized for Western blotting and IHC.

Antibody/Dilution	Source
Bad 1:100	ProSci Inc. 3343
Bid 1:100	ProSci Inc. 3353
Bim 1:100	ProSci Inc. 2065
Noxa 1:100	ProSci Inc. 2437
Puma 1:100	ProSci Inc. 3041
Bik 1:100	ProSci Inc. 3891
DP5/Hrk 1:100	ProSci Inc. 3771
GFAP 1:100	Abcam ab464-100
ChAT 1:250	Chicken anti-mouse monoclonal, Chemicon AB15468
BNip3L 1:100	Abcam ab38621
Cy3-conjugated secondary (1:500)	Donkey anti-rabbit or anti-mouse, Jackson ImmunoResearch, 711165152/715165150
FITC-conjugated secondary (1:500)	Donkey anti-rabbit or anti-mouse, Jackson ImmunoResearch, 711095152/715095150
Cy3-conjugated secondary (1:500)	Donkey anti-chicken, Jackson ImmunoResearch 703165155
DP5/Hrk (Western Blotting; 1:700)	Abcam ab45419
BNip3L (Western Blotting; 1:250)	Abcam ab38621
β-tubulin (Western Blotting; 1:200)	Sigma T4026
COX-IV (Western Blotting; 1:1000)	Cell Signaling 4884


### Western Blot Analysis

Lumbar spinal cord tissue samples were lysed using the following buffer; 20 mM HEPES (pH 7.4), 1% Triton X-100, 50 mM NaCl, 1 mM EGTA, 5 mM β-glycerophosphate, 30 mM sodium pyrophosphate, 100 μM sodium orthovanadate, 10 μg/ml leupeptin and 10 μg/ml aprotinin, by homogenization 20 times with a (glass/glass) tight dounce homogenizer. Samples were then centrifuged at 10,000 rpm for 2 min and supernatant was collected. Lysates were subjected to sodium dodecyl sulfate-polyacrylamide gel electrophoresis (SDS-PAGE) on 12.5% or 15% polyacrylamide gels. Resolved proteins were transferred to polyvinylidene difluoride membranes and processed for immunoblot analysis as previously described (Loucks et al., 2006). Antibodies utilized for Western blotting are listed in **Table 1**.

### Statistical Analysis and Quantification of Data

Each BH3-only protein was stained in end-stage G93A mutant animals and age-matched WT animals. Images captured from IHC of BH3-only proteins were quantified by counting total numbers of astrocytes and motor neurons which stained positively for BH3-only proteins in lumbar spinal cord sections. Large alpha motor neurons were identified by size or immunoreactivity for ChAT. Reactive astrocytes were identified by their star-like morphology or immunoreactivity for GFAP. Five 40X fields were captured and quantified for each BH3-only protein analyzed per animal. The total number of animals analyzed per BH3-only protein is given in the figure legends.

## Results and Discussion

### IHC Analysis of BH3-Only Protein Expression in Lumbar Spinal Cord Motor Neurons

**FIGURE 1:**
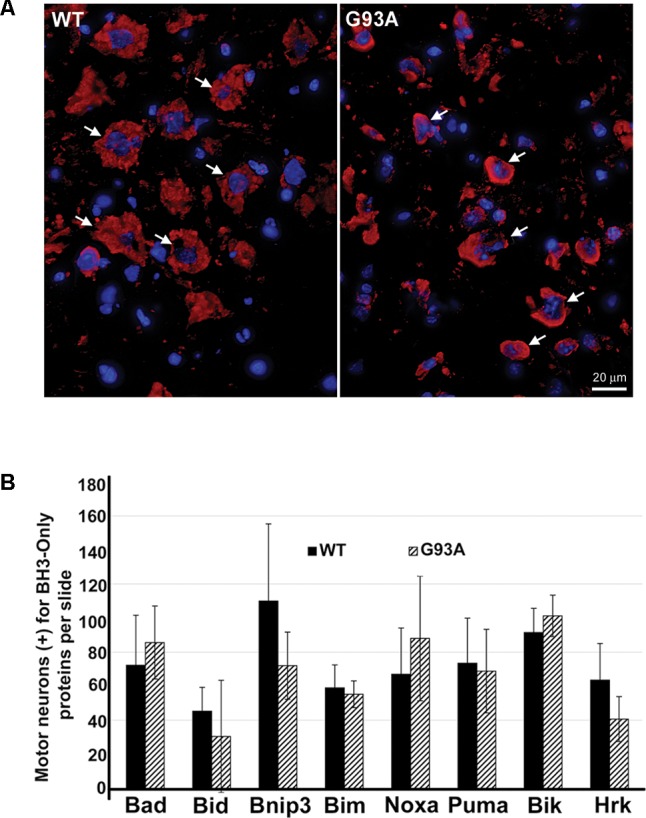
IHC of WT and G93A mutant SOD1 lumbar spinal cord for BH3-only protein expression in motor neurons. **(A)** IHC of Bik expression in motor neurons of G93A and WT lumbar spinal cord tissue. Lumbar spinal cord was sagittally cut 8 μm thick onto standard glass slides. Slides were stained and imaged for Bik (Cy3, red) and DAPI (blue). Arrows point to large Bik-positive motor neurons. The image shown is representative of the results observed with other BH3-only proteins, Bim, Noxa, Puma, and Bad. **(B)** Quantification of BH3-only positive motor neurons. Data shown represent the mean ± SEM for the number of BH3-only positive motor neurons quantified from five 40X fields per animal. Mean and SEM were calculated from *N* = 5–7 WT and G93A mice per BH3-only protein. When analyzed by unpaired *t*-test, no significant differences in the number of BH3-only positive motor neurons were found between WT and G93A mutant SOD1 lumbar spinal cord sections for any of the BH3-only proteins analyzed.

Using IHC, we analyzed BH3-only protein expression in end-stage G93A mutant SOD1 mice compared to age-matched WT mice. **Figure 1A** shows lumbar spinal cord sections representative of WT and G93A mutant SOD1 mice stained for Bik (red) and DAPI (blue). Large alpha motor neurons appeared to stain consistently positive for Bik (arrows), regardless of the genotype of the mice. It was notable that motor neurons in end-stage G93A mutant spinal cord appeared to have smaller soma than those in WT spinal cord, indicative of motor neuron degeneration in the ALS mice. However, no discernible differences were observed in either the intensity of Bik protein expression or its localization between WT and G93A mutant SOD1 end-stage mice. **Figure 1A** is representative of similar results found for Bim, Noxa, Puma and Bad (data not shown). The expression of these additional BH3-only proteins in lumbar spinal cord sections of both WT and end-stage G93A mutant SOD1 mice was clearly evident in the large α-motor neurons; however, no apparent difference in either expression or localization of these BH3-only proteins were observed in mutant mice. **Figure 1B** shows quantification of the average number of motor neurons (per five 40X fields) from lumbar spinal cord of WT and end-stage G93A mutant SOD1 mice which stained positively for BH3-only proteins (*N* = 5–7 mice per genotype and per BH3-only protein). No significant differences were observed for BH3-only protein expression in motor neurons between end-stage G93A mutant SOD1 and WT lumbar spinal cord sections.

### Specific Staining for Bid in Reactive Astrocytes of End-Stage Mutant SOD1 Spinal Cord

**FIGURE 2:**
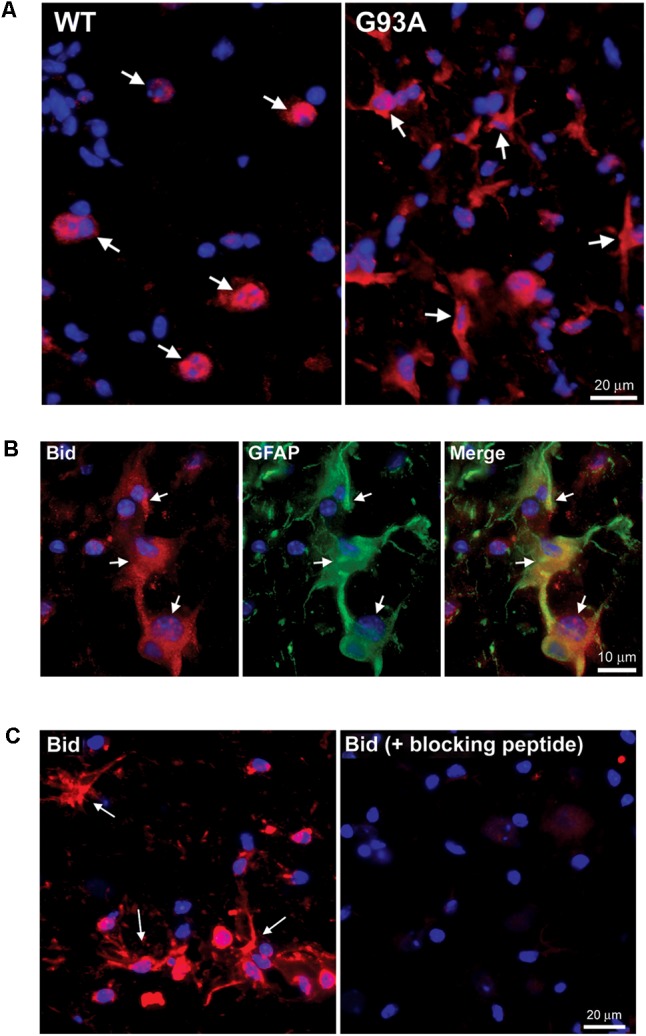
Specific expression of Bid in astrocytes of end-stage G93A mutant SOD1 mouse spinal cord. **(A)** IHC of Bid (Cy3, red) in WT and end-stage G93A lumbar spinal cord tissue. Arrows point to Bid-positive motor neurons in the WT panel. Arrows point to Bid-positive cells with a “star-like” morphology in the G93A mutant SOD1 panel. **(B)** Bid (Cy3, red) contained with the astrocyte marker GFAP (FITC, green) to confirm Bid-positive cells in the G93A mutant SOD1 lumbar spinal cord as astrocytes. **(C)** Primary Bid antibody was pre-incubated with a blocking peptide prior to IHC to show its specificity for Bid. Arrows point to Bid-positive astrocytes in the Bid panel. Staining is significantly diminished with addition of the Bid blocking peptide. In **A–C** nuclei are stained with DAPI (blue).

Upon IHC staining for BH3-only proteins, we observed a cell type distinct from motor neurons that stained strongly positive for specific BH3-only proteins only in the end-stage G93A mutant SOD1 lumbar spinal cord sections. In **Figure 2A**, arrows in the WT panel indicate Bid-positive motor neurons, however; the arrows in the G93A panel highlight Bid-positive cells with a unique morphology clearly distinct from that of motor neurons. The star-like morphology of these Bid-positive cells observed in G93A spinal cord was indicative of reactive astrocytes. In contrast, Bid protein expression in WT spinal cord was largely restricted to cells with morphology typical of large alpha motor neurons. We next co-stained end-stage, G93A mutant SOD1 lumbar spinal cord sections with Bid (red) and glial fibrillary acidic protein (GFAP, green), an astrocyte marker. The Merge panel shown in **Figure 2B** displays co-localization (shown in yellow) between the BH3-only protein Bid and GFAP, demonstrating that the Bid-positive cells detected in end-stage G93A mutant SOD1 lumbar spinal cord are indeed reactive astrocytes. To verify the specificity of the Bid primary antibody, we pre-incubated the primary antibody solution with a blocking peptide specific for the Bid antigen. In **Figure 2C**, the left panel shows positive staining for Bid (red) in end-stage G93A mutant SOD1 lumbar spinal cord with at least three astrocytes displaying Bid expression (arrows). However, pre-incubation with the blocking peptide (right panel) significantly diminishes the Bid immunoreactivity in another spinal cord section from the same end-stage mouse, indicating the specificity of the Bid antibody.

### Specific Staining for Hrk and BNip3L in Reactive Astrocytes of End-Stage G93A SOD1 Mouse Spinal Cord

**FIGURE 3:**
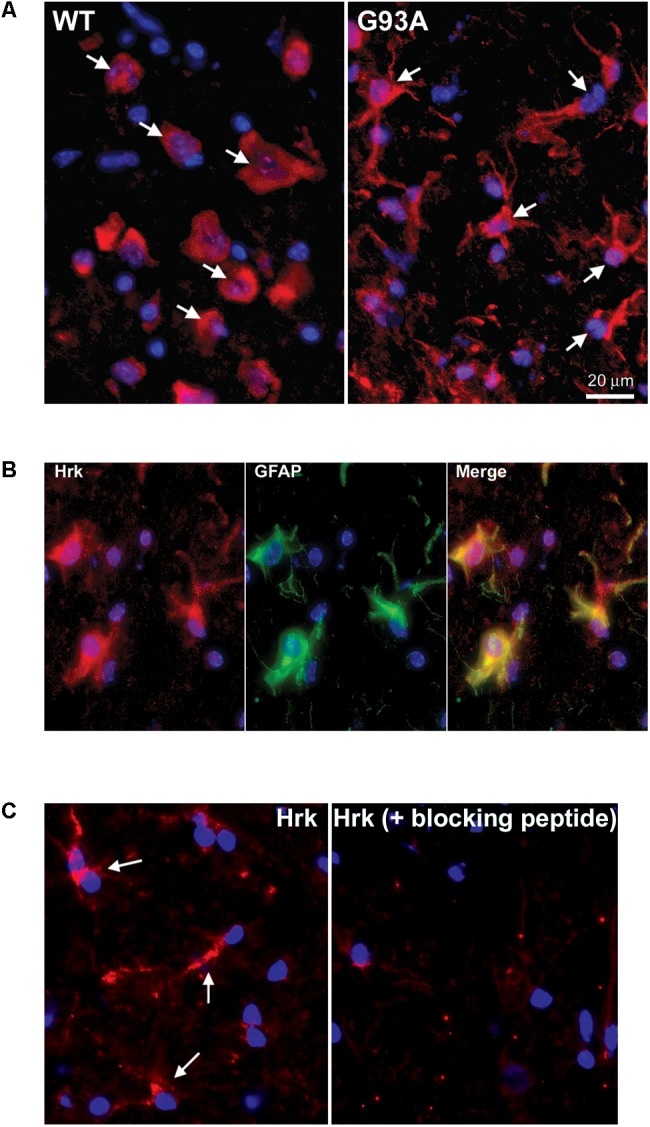
Specific expression of Hrk in astrocytes of end-stage G93A mutant SOD1 mouse spinal cord. **(A)** Lumbar spinal cord sections were stained for Hrk (Cy3, red) and DAPI (blue). Arrows point to Hrk-positive motor neurons in the WT panel. Arrows point to Hrk-positive cells with a “star-like” morphology in the G93A mutant SOD1 panel. **(B)** IHC of Hrk co-stained with the astrocyte marker GFAP in end-stage G93A mutant SOD1 lumbar spinal cord tissue. Slides were stained with Hrk (Cy3, red), DAPI (blue), and GFAP (FITC, green). **(C)** Primary Hrk antibody was pre-incubated with a blocking peptide prior to IHC to show its specificity for Hrk. Arrows point to Hrk-positive astrocytes in the Hrk panel. Staining is significantly diminished with the addition of the Hrk blocking peptide.

**Figure 3A** shows end-stage G93A mutant SOD1 versus WT lumbar spinal cord sections stained for Hrk (red) and DAPI (blue). Arrows indicate large, Hrk-positive motor neurons in the WT panel, whereas in the G93A panel, arrows identify Hrk-positive cells with a star-like morphology clearly distinct from that of motor neurons. The star-like morphology of the Hrk-positive cells in the end-stage G93A mutant SOD1 mouse spinal cord is similar to the results observed for Bid in **Figure 2**. Lumbar spinal cord sections for end-stage G93A mutant SOD1 mice were co-stained for Hrk (red), GFAP (green), and DAPI (blue) in **Figure 3B**. The Merge panel demonstrates co-localization (yellow) between Hrk and GFAP, indicating that the Hrk-positive cells in the G93A spinal cord sections are indeed astrocytes. As shown in **Figure 2** for Bid, Hrk staining of end-stage astrocytes was significantly diminished upon pre-incubation of the primary antibody solution with a blocking peptide, indicating the specificity of the Hrk antibody (**Figure 3C**).

**FIGURE 4:**
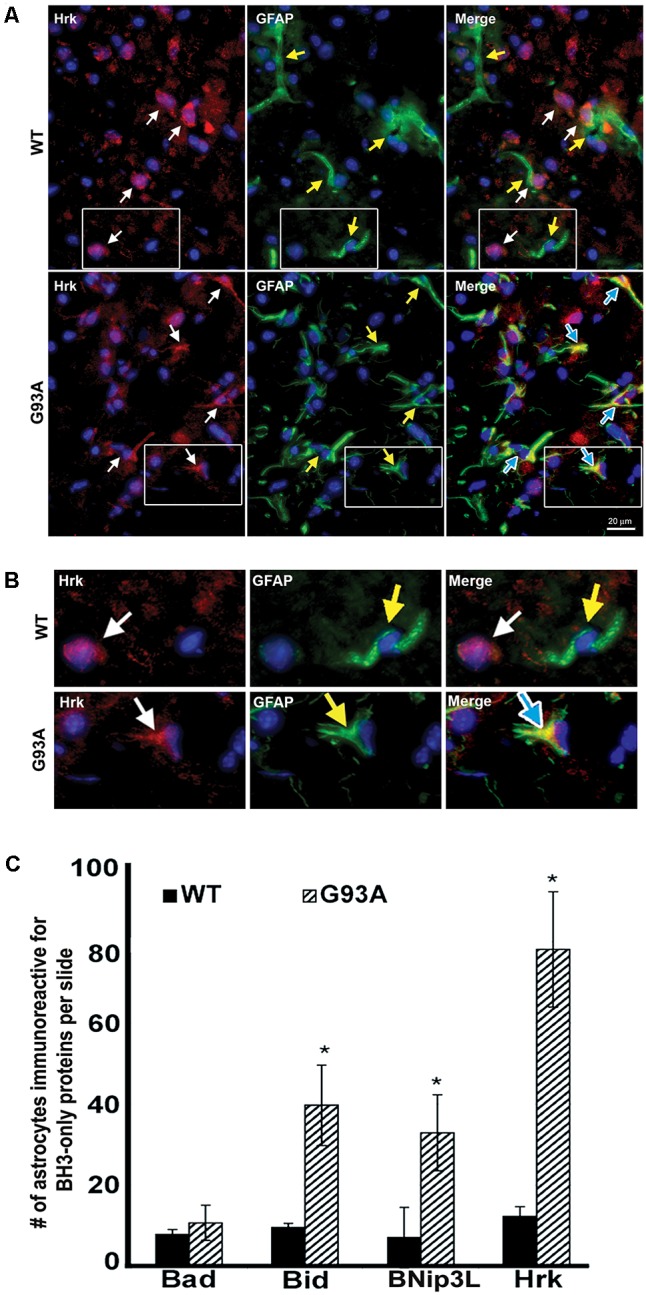
Astrocytes from end-stage G93A mutant SOD1 mouse spinal cord show enhanced expression of Bid, BNip3L, and Hrk. **(A)** Lumbar spinal cord sections were stained for Hrk (Cy3, red), DAPI (blue) and GFAP (FITC, green). White arrows indicate Hrk-positive cells in the WT and G93A Hrk panels (left), respectively. Yellow arrows indicate GFAP-positive cells in the WT and G93A GFAP panels (middle), respectively. In the WT Merge panel (upper right) little-to-no co-localization of Hrk and GFAP was observed. In contrast, the G93A Merge panel (lower right) shows substantial Hrk and GFAP co-localization (yellow staining) indicated by blue arrows. **(B)** Areas demarcated by boxes are enlarged 300% to show co-localization. **(C)** Number of BH3-only-positive astrocytes in WT versus G93A mutant SOD1 lumbar spinal cord tissue samples stained for Bad, Bid, BNip3L, and Hrk, analyzed by one-way ANOVA with a *post hoc* Dunnett’s test (^∗^*p* < 0.05 vs. WT). Data shown represent the mean ± SEM for the number of BH3-only positive astrocytes quantified from five 40X fields per animal. Mean and SEM were calculated from *N* = 4–5 WT and G93A mice per BH3-only protein.

To further evaluate the specificity of astrocyte staining for Hrk in end-stage G93A mutant SOD1 mice, co-staining of GFAP and Hrk was compared between WT and mutant spinal cord. In **Figure 4A**, the WT panels (top row) show Hrk (red), GFAP (green), and Merged images. White arrows indicate Hrk-positive cells and yellow arrows indicate GFAP-positive cells. In the Merge panel, little-to-no co-staining was observed in the WT lumbar spinal cord for Hrk and GFAP. In contrast, significant co-localization was observed between GFAP and Hrk in G93A lumbar spinal cord sections. In the G93A panels (bottom row), Hrk-positive cells (red) are indicated by white arrows, GFAP-positive cells (green) are indicated by yellow arrows, and the Merge image shows pronounced co-localization of Hrk and GFAP (yellow) identified by blue arrows (**Figure 4A**). Enlargement of specific cells shows the specificity of the co-localization of Hrk and GFAP in G93A sections, absent from WT sections (**Figure 4B**). These results suggest that Hrk is specifically upregulated in G93A mutant SOD1 astrocytes compared to WT in lumbar spinal cord.

Further analysis of lumbar spinal cord sections from four WT and five G93A mutant mice indicated a significant difference in the average number of astrocytes staining positively for Bid, BNip3L or Hrk. Lumbar spinal cord sections were stained for the BH3-only proteins Bad, Bid, BNip3L or Hrk and the number of astrocytes staining positively for these BH3-only proteins (per five 40X fields) was statistically analyzed. No significant difference was found in the number of astrocytes staining positively for Bad in WT versus end-stage G93A lumbar spinal cord sections (**Figure 4C**). In contrast, significant differences in the number of astrocytes staining positively in WT versus end-stage G93A mutant SOD1 lumbar spinal cord were observed for the BH3-only proteins Bid, BNip3L, and Hrk (*p* < 0.05 vs. WT, **Figure 4C**). These findings support the conclusion that Bid, BNip3L and Hrk are being selectively upregulated in astrocytes of end-stage G93A mutant SOD1 lumbar spinal cord when compared to age-matched WT controls.

### Specificity of Hrk Expression in End-Stage Spinal Cord Astrocytes

**FIGURE 5:**
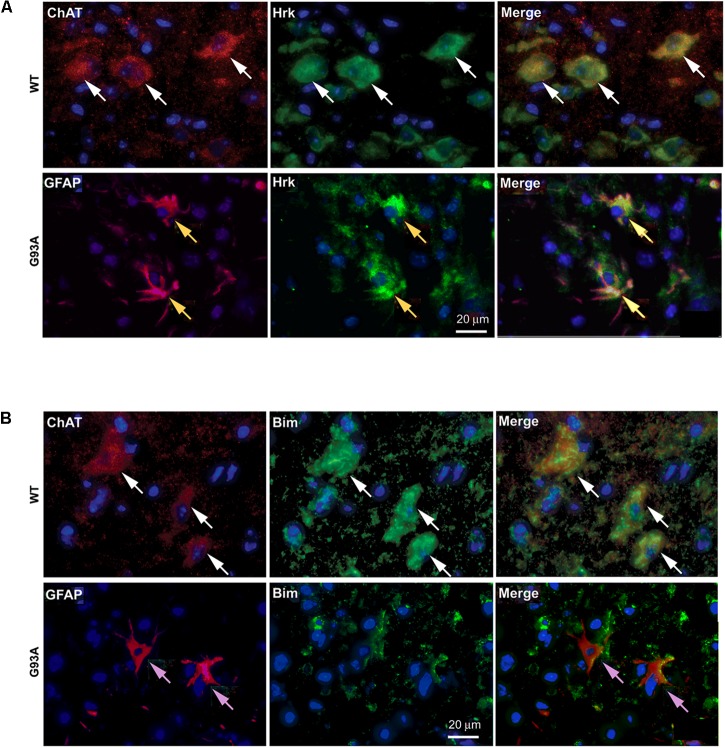
Cell type-specific localization of BH3-only proteins, Hrk and Bim, in WT versus G93A mutant SOD1 lumbar spinal cord. **(A)** WT panels (top row) show Hrk (FITC, green), ChAT (Cy3, red), and DAPI (blue). Merge panel shows co-localization (yellow) of ChAT and Hrk in WT lumbar spinal cord sections (white arrows). G93A panels (bottom row) show Hrk (FITC, green), GFAP (Cy3, red), and DAPI (blue). Merge panel shows co-localization (yellow) of Hrk and GFAP in G93A mutant SOD1 lumbar spinal cord sections (yellow arrows). **(B)** WT panels (top row) show ChAT (Cy3, red), Bim (FITC, green), and DAPI (blue). Merge panel shows ChAT and Bim co-localization (yellow) in WT lumbar spinal cord sections (white arrows). G93A panels (bottom row) show Bim (FITC, green), GFAP (Cy3, red), and DAPI (blue). Merge panel shows little-to-no co-localization of Bim and GFAP in G93A mutant SOD1 lumbar spinal cord sections (pink arrows).

To further show specificity of Hrk expression in G93A mutant SOD1 spinal cord astrocytes, co-localization was assessed between either Hrk or Bim and ChAT or GFAP in both WT and end-stage sections. In **Figure 5A**, the WT panels (top row) show staining for ChAT (red) and Hrk (green). We observed co-localization (shown in yellow) between Hrk and ChAT in the WT lumbar spinal cord sections (Merge), indicating that Hrk is largely expressed in motor neurons of WT spinal cord. The G93A panels (bottom row) display end-stage lumbar spinal cord sections stained for Hrk (green) and GFAP (red). The G93A Merge panel shows significant co-localization (yellow) between Hrk and GFAP, consistent with astrocyte-specific expression of this BH3-only protein in end-stage mice. Similar results were observed for Bid and BNip3L (data not shown). A similar experiment is shown in **Figure 5B** for the BH3-only protein Bim. Here, we show WT lumbar spinal cord sections (top row) stained for ChAT (red) and Bim (green). The WT Merge panel shows substantial co-localization between Bim and ChAT, similar to the results found for Hrk in WT animals. The G93A panels (bottom row) show staining for Bim (green) and GFAP (red). In contrast to the results observed for Hrk in G93A mutant mice, no significant co-localization was observed between Bim and GFAP in end-stage spinal cord (**Figure 5B**). The results for Bim were consistent with those observed for Bad, Bik, Noxa and Puma (data not shown). Therefore, the co-localization between GFAP and the BH3-only proteins Hrk, Bid, and Bnip3L is specific for end-stage, G93A mutant SOD1 mouse spinal cord.

**FIGURE 6:**
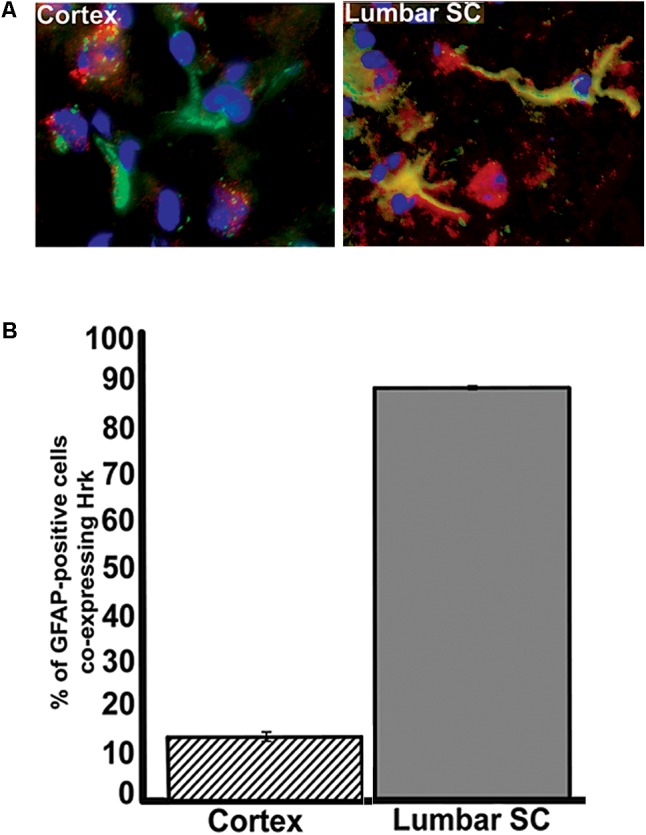
Analysis of Hrk and GFAP co-localization in cerebral cortex and lumbar spinal cord tissue of end-stage G93A mutant SOD1 mice. **(A)** Transverse sections of cortex and sagittal sections of lumbar spinal cord were co-stained for Hrk (Cy3, red), GFAP (FITC, green), and DAPI (blue). **(B)** Quantification of co-staining for Hrk and GFAP in cerebral cortex and lumbar spinal cords of end-stage G93A mutant SOD1 mice. Two sections of each tissue type were analyzed from two different G93A mutant SOD1 end-stage animals. Co-localization of Hrk and GFAP appears specific to lumbar spinal cord tissue of G93A mutant SOD1 end-stage animals.

To further show specificity of Hrk expression in G93A mutant SOD1 spinal cord astrocytes, we next compared the fraction of GFAP-expressing astrocytes that co-expressed Hrk in end-stage spinal cord to sections of end-stage cerebral cortex, a brain regions that are largely unaffected in ALS (with the exception of the motor cortex). Sections of cortex and spinal cord from an individual end-stage G93A mutant SOD1 mouse were co-stained for Hrk (red), DAPI (blue), and GFAP (green). **Figure 6A** illustrates that co-localization of Hrk and GFAP (shown in yellow) was only found to be significantly present in astrocytes of lumbar spinal cord sections, but not sections from cortex. **Figure 6B** shows the percentage of GFAP-positive astrocytes co-stained for Hrk in the cortex and lumbar spinal cord from two end-stage G93A mutant SOD1 mice. The lumbar spinal cord sections of end-stage mice showed that 88% ± 0.4% of astrocytes co-stained for Hrk, while the cortex were co-stained at 14% ± 1%, respectively. These data strongly suggest that Hrk upregulation in astrocytes of G93A mutant SOD1 animals is selective for the spinal cord over the cortex. However, we have not examined brain stem nuclei which may also be affected in a similar manner to the spinal cord.

### Western Blotting of WT and End-Stage Spinal Cord Lysates for Hrk and BNip3L

**FIGURE 7:**
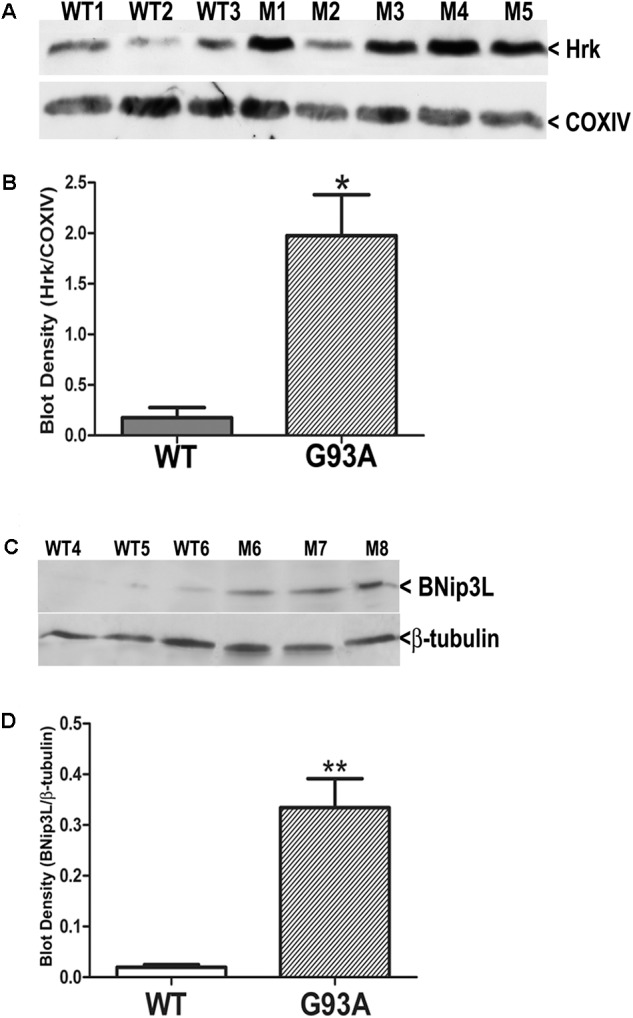
Western blot analysis of Hrk and BNip3L expression in WT and G93A mutant SOD1 lumbar spinal cord tissue. **(A)** Hrk protein expression in 3 WT and 5 G93A mutant (M) SOD1 animals. COX-IV serves as a loading control. **(B)** Densitometry of Hrk bands normalized to COX-IV loading control and analyzed by unpaired *t*-test: WT versus G93A, ^∗^*p* < 0.05. **(C)** BNip3L protein expression in three WT and three G93A mutant SOD1 animals. β-tubulin serves as a loading control. **(D)** Densitometry of BNip3L bands normalized to β-tubulin loading control, and analyzed by unpaired *t*-test: WT versus G93A, ^∗∗^*p* < 0.01. WT, wild type mouse; M, SOD1 mutant mouse.

Finally, we utilized western blotting to further confirm the upregulation of Hrk and BNip3L in end-stage G93A mutant SOD1 lumbar spinal cord lysates. **Figure 7A** shows Hrk western blotting for 3 WT and 5 end-stage G93A mutant SOD1 mice. A marked and consistent increase in Hrk expression was observed in 4 of 5 end-stage G93A mutant SOD1 lumbar spinal cord tissue samples. When normalized to the loading control, Cox IV, a significant increase in Hrk expression in end-stage spinal cord was confirmed using densitometry (**Figure 7B**). We next examined BNip3L in three WT and three end-stage, G93A mutant SOD1 lumbar spinal cord samples (**Figure 7C**). A consistent and significant increase in BNip3L expression was observed in end-stage spinal cord when band densities were normalized to the loading control, β-tubulin (**Figure 7D**). Thus, the enhanced expression of the specific BH3-only proteins, Hrk and BNip3L, in reactive astrocytes of end-stage G93A mutant SOD1 mouse spinal cord was also detectable by western blotting as significant increases in whole spinal cord lysates. In contrast, we could not detect any significant increase in Bid expression by western blotting of whole spinal cord lysates (data not shown). This may reflect a relatively high expression of Bid in motor neurons of WT mice, which may mask the enhanced expression of this BH3-only protein in reactive astrocytes of end-stage mice.

## Conclusion

In the current study, IHC analysis demonstrated basal expression of several BH3-only family members within spinal cord motor neurons; however, neither the qualitative levels of expression nor the relative numbers of immunoreactive motor neurons varied significantly between WT and end-stage G93A mutant SOD1 mice. In marked contrast to motor neurons, we found that a specific subset of BH3-only proteins, including Bid, Hrk, and BNip3L, are selectively upregulated in reactive astrocytes of end-stage G93A mutant SOD1 mouse spinal cord. Our evidence supporting this conclusion is as follows: (1) IHC analysis of BH3-only proteins in lumbar spinal cord sections showed that Bid, Hrk, and BNip3L were highly expressed in GFAP-positive, reactive astrocytes of end-stage G93A mice, while in age-matched WT mice these proteins were expressed predominantly in motor neurons; (2) blocking peptides to Bid and Hrk significantly diminished the staining of reactive astrocytes for these BH3-only proteins in end-stage G93A mice; (3) we detected a statistically significant increase in the numbers of reactive astrocytes which were positive for Bid, Hrk, or BNip3L in end-stage G93A mice while the numbers of Bad-positive astrocytes did not differ significantly from WT mice; (4) additional BH3-only family members (e.g., Bim) did not show any enhanced expression in astrocytes of end-stage G93A mice (data not shown); (5) the percentage of GFAP-positive astrocytes co-expressing Hrk in end-stage G93A mice was nearly 90% in lumbar spinal cord but less than 20% in cerebral cortex and cerebellum (areas largely unaffected in ALS); and (6) Western blotting for Hrk and BNip3L in whole spinal cord lysates demonstrated large increases in the expression of these BH3-only proteins in end-stage G93A mice when compared to age-matched WT mice. Together, these data convincingly demonstrate a unique and specific upregulation of the BH3-only cohort, Bid, Hrk, and BNip3L, in reactive astrocytes of end-stage G93A mutant SOD1 mouse spinal cord.

The molecular mechanisms which underlie reactive astrogliosis stem from a complex interplay of extracellular and intracellular signals that culminate in profound changes in astrocyte gene expression, ultimately impacting astrocyte function, morphology, hypertrophy, and proliferation (reviewed by Sofroniew and Vinters, 2010). The potential pathological roles of reactive astrocytes in neurodegeneration are varied and may involve loss of essential normal astrocyte functions or gain of reactive astrocyte toxic functions (Sofroniew and Vinters, 2010). In the context of ALS, both of these mechanisms have been postulated to contribute to the pathological functions of reactive astrocytes during motor neuron disease (Ilieva et al., 2009; Vargas and Johnson, 2010; Yang et al., 2011; Hall et al., 2017). The most prominent example of loss of normal astrocyte function in ALS is the marked downregulation of the astrocyte-specific glutamate transporter, GLT-1, in spinal cord of sporadic ALS patients and G93A mutant SOD1 transgenic mice and rats (Rothstein et al., 1992, 1995; Dunlop et al., 2003; Guo et al., 2010). This loss of GLT-1 transporter activity leads to significant deficits in glutamate uptake by astrocytes which contribute to the excitotoxic injury of motor neurons in ALS. In regards to gain of toxic functions, reactive astrocytes often display a neuroinflammatory phenotype characterized by enhanced production of reactive oxygen and nitrogen species and release of excitotoxic glutamate or pro-inflammatory cytokines and chemokines. Consistent with this mechanism playing a key role in the pathology of ALS, astrocytes expressing mutant forms of SOD1 directly induce motor neuron death in co-culture systems *in vitro*, and this effect appears to be mediated by soluble factors or free radicals secreted by these reactive glial cells (Nagai et al., 2007; Bilsland et al., 2008). Additionally, reactive astrocytes may induce protein accumulation by releasing transforming growth factor β1 (TGF-β1), which disrupts autophagy in motor neurons through the mammalian target of rapamycin (mTOR) pathway (Tripathi et al., 2017).

Given the importance of reactive astrocytes in causing motor neuron death in ALS, it is not surprising that recent strategies have focused on manipulating these glial cells to slow disease progression. For instance, focal transplantation of normal astrocyte precursors juxtaposed to the cervical spinal cord respiratory motor neuron pools significantly extended survival of G93A mutant SOD1 transgenic rats (Lepore et al., 2008b). Another strategy employed astrocyte-specific expression of the Nrf2 transcription factor in transgenic mice using a GFAP promoter (Vargas et al., 2008). Astrocytes co-expressing mutant SOD1 and Nrf2 did not induce death of co-cultured motor neurons, an effect that was dependent on glutathione release from Nrf2-overexpressing astrocytes. Moreover, crossing of mutant SOD1 mice with GFAP-Nrf2 transgenic mice delayed disease onset and significantly extended survival. Thus, manipulating the astrocyte population to decrease the prevalence of the neuroinflammatory phenotype of these glial cells can be successfully utilized to reduce motor neuron death and slow disease progression in ALS. Recently, it was shown that ephrin type-B receptor 1 (EphB1) was upregulated in injured motor neurons causing activation of astrocytes through EphB1 – stimulation of signal transducer and activator of transcription-3 (STAT3) pathway, identifying a potential neuroprotective astrocyte response, which is disrupted in ALS (Tyzack et al., 2017). Therefore, elucidating the molecular events that induce development of the astrocyte neuroinflammatory phenotype may provide new therapeutic targets for intervention in ALS or other neurodegenerative disorders involving reactive astrogliosis.

As mentioned above, astrocytes expressing various mutant forms of SOD1 display a neuroinflammatory phenotype and directly induce motor neuron death in co-culture systems (Nagai et al., 2007; Bilsland et al., 2008). Interestingly, even astrocytes isolated from G93A mutant SOD1 transgenic mouse (P7) neonates display enhanced levels of basal or tumor necrosis factor alpha-stimulated pro-inflammatory eicosanoids and reactive oxygen and nitrogen species (Hensley et al., 2006). This neuroinflammatory phenotype was observed in astrocytes *in vitro* despite the fact that no evidence of neuroinflammation was observed *in vivo* in 7-day-old G93A mutant SOD1 mice. These data suggest that astrocytes expressing mutant SOD1 are “primed” to become reactive or neuroinflammatory in phenotype.

Our findings suggest the intriguing possibility that the expression of a specific subset of BH3-only proteins (Bid, Hrk, and BNip3L) may contribute to the maintenance of this neuroinflammatory phenotype in astrocytes expressing G93A mutant SOD1. Based on our findings, the high level of expression of these BH3 only proteins do not appear to induce apoptosis in reactive astrocytes as no significant nuclear condensation or fragmentation was observed in GFAP-positive cells which were also immunoreactive for Bid, Hrk, or BNip3L. In addition, the morphology looks entirely healthy. We did not directly assess apoptosis with TUNEL staining or active caspase-3 staining, however, a previous study also showed upregulation of Bid in reactive astrocytes in mutant mice and did not demonstrate any apoptosis in these astrocytes by caspase-3 and PARP cleavage (König et al., 2014). Therefore, an obvious question that arises is – what is the *non-apoptotic* role of these BH3-only proteins in reactive astrocytes? Several studies have shown that various BH3-only proteins display a number of key non-apoptotic functions in diverse cell types (reviewed by Lomonosova and Chinnadurai, 2008). For example, Bad has been shown to induce cell cycle progression in fibroblasts subjected to growth arrest conditions such as low serum or confluence (Chattopadhyay et al., 2001). Similarly, Bad overexpression enhances cell cycle progression and interleukin-2 production in activated T cells (Mok et al., 1999). In addition, several BH3-only proteins have been shown to regulate mitochondrial and endoplasmic reticulum function. Bad is an integral component of a mitochondrial protein complex that regulates glucokinase activity and mitochondrial respiration (Danial et al., 2003). Bid displays lipid transfer activity and influences the transport and recycling of mitochondrial phospholipids (Esposti et al., 2001). The BNip3L relative, BNip1, is a component of a SNARE complex that regulates the structure of the endoplasmic reticulum network (Nakajima et al., 2004). These studies support the hypothesis that BH3-only proteins may have novel *non-apoptotic* functions that contribute to the neuroinflammatory phenotype of reactive astrocytes expressing mutant SOD1.

One mechanism by which BH3-only proteins might influence the neuroinflammatory phenotype of astrocytes in ALS is via an enhancement of cell cycle progression and as a result, increases in glial proliferation. However, strategies which selectively ablated proliferating astrocytes in the G93A mutant SOD1 mouse did not significantly affect disease onset, survival, or the overall extent of astrogliosis in this ALS model (Lepore et al., 2008a). Given the non-apoptotic functions of BH3-only proteins at mitochondria discussed above, it is tempting to speculate that BH3-only proteins in reactive astrocytes may induce mitochondrial dysfunction in these glial cells. Since the astrocytes observed in our study showed high levels of expression of specific BH3-only proteins without detectable apoptosis, the predicted mitochondrial dysfunction induced by these BH3-only proteins would have to be intrinsically non-toxic to astrocytes and yet, sufficient to trigger death in neighboring motor neurons. Interestingly, a study by Cassina et al. (2008) demonstrated that astrocytes expressing G93A mutant SOD1 displayed deficits in mitochondrial respiration and enhanced oxidative and nitrosative stress. Pre-incubation of these G93A astrocytes with mitochondrial-targeted antioxidants protected co-cultured motor neurons from death indicating that mitochondrial dysfunction in mutant astrocytes indeed promotes the death of neighboring motor neurons. Recently, it was shown that Bid is critical for pro-inflammatory signaling through the activation of the transcription factor nuclear factor-κB in response to pro-inflammatory stimuli in the G93A mutant SOD1 mouse model of ALS (König et al., 2014). However, the potential of Hrk and BNip3L in the neuroinflammatory transition of astrocytes in ALS have not yet been explored. Future studies will be required to define the precise roles of the BH3-only proteins, Bid, Hrk, and BNip3L, in reactive astrocytes of end-stage G93A mutant SOD1 mice. Identification of the possible *non-apoptotic* functions of these BH3-only proteins in reactive astrocytes may reveal novel pathways that could be targeted to diminish astrogliosis and slow disease progression in ALS.

## Author Contributions

ND, WS, AA, JG, RB, and HW: Substantial contributions to the design and acquisition of the work. Interpretation and analysis of work. Drafting and editing of the manuscript. DL: Intellectual content, concept and design of work. Drafting, editing, and final approval of the manuscript.

## Conflict of Interest Statement

The authors declare that the research was conducted in the absence of any commercial or financial relationships that could be construed as a potential conflict of interest.
